# Recent Advances in the Treatment of ST-Segment Elevation Myocardial Infarction

**DOI:** 10.6064/2012/683683

**Published:** 2012-09-16

**Authors:** Mun K. Hong

**Affiliations:** ^1^Cardiac Catheterization Laboratory and Interventional Cardiology, St. Luke's-Roosevelt Hospital Center, 1111 Amsterdam Avenue, New York, NY 10025, USA; ^2^Columbia University College of Physicians and Surgeons, 630 W. 168th St., New York, NY 10032, USA

## Abstract

ST-segment elevation myocardial infarction (STEMI) represents the most urgent condition for patients with coronary artery disease. Prompt diagnosis and therapy, mainly with primary angioplasty using stents, are important in improving not only acute survival but also long-term prognosis. Recent advances in angioplasty devices, including manual aspiration catheters and drug-eluting stents, and pharmacologic therapy, such as potent antiplatelet and anticoagulant agents, have significantly enhanced the acute outcome for these patients. Continuing efforts to educate the public and to decrease the door-to-balloon time are essential to further improve the outcome for these high-risk patients. Future research to normalize the left ventricular function by autologous stem cell therapy may also contribute to the quality of life and longevity of the patients surviving STEMI.

## 1. Introduction

ST-segment elevation myocardial infarction (STEMI) accounts for approximately 30–45% of an estimated 1.5 million hospitalizations for acute coronary syndromes annually in the USA [1]. STEMI results primarily from sudden-onset plaque rupture and complete occlusion of a coronary artery [2]. Therefore, STEMI represents the most severe form of acute coronary syndromes and requires immediate therapy. There have been many recent advances in the treatment of STEMI, ranging from pharmacologic to device therapy. These advances have resulted in improved outcomes for the patients experiencing STEMI [3, 4]. In-hospital mortality from STEMI decreased steadily in the USA in all groups between 1997 and 2006, except for men <55 years of age [4]. Thus, there is still much more work to be done, especially the prevention of its occurrence in young men due to the unpredictable timing and relatively high risk of sudden death [5]. In addition, complete myocardial perfusion to improve left ventricular function and survival is essential but may not be achieved in many patients due to multifactorial reasons [6–12] and needs continued research for better long-term outcome.

## 2. Reperfusion Therapies

The most important therapy for STEMI patients is the prompt reestablishment of antegrade flow. The earlier and the more complete the reperfusion, the greater the myocardial salvage and preservation of left ventricular function, the most important prognostic factor for long-term survival. There are pharmacologic and mechanical reperfusion therapies. Randomized trials have conclusively established primary angioplasty with stents as the optimal therapy for these patients, as primary percutaneous coronary intervention (PCI) provides >90% TIMI 3 flow in the infarct-related vessel compared with only approximately 50% with thrombolytic therapy, thereby improving myocardial salvage and survival, as well as preventing recurrent ischemia and reinfarction by definitively treating the severe stenosis that can result even after a successful thrombolysis [13]. Needless to say, primary PCI should be performed at centers with high volumes by experienced interventional cardiologists. The National Registry of Myocardial Infarction (NRMI) showed that mortality was significantly reduced at hospitals with greater monthly primary PCI procedures versus the lower volume centers [14]. Subsequent NRMI-2 and NRMI-3 registries confirmed the importance of high PCI volumes as an important predictor of mortality benefit compared with thrombolytic therapy, which was not seen at low-volume primary PCI hospitals [15]. In addition to the hospital volume, annual individual primary PCI volume was found to correlate significantly with in-hospital survival following primary PCI [16]. When these requirements are met, primary PCI reduced mortality by 25%, reinfarction by 64%, intracranial hemorrhage by 95%, and stroke by 53% versus thrombolytic therapy. Door-to-balloon (D2B) time for primary angioplasty has been shown to be very important in salvaging ischemic myocardium and improving survival [17]. Metaregression analysis of 20 summary data points from the randomized comparative trials between primary PCI and thrombolytic therapy showed that D2B time of >90 minutes could eliminate the mortality benefit of primary PCI versus thrombolytic therapy, suggesting the importance of achieving as rapid a D2B time as possible, with recommended D2B time <90 minutes [18]. There are many potential ways to improve the D2B time. These strategies have included emergency medicine physicians activating the catheterization laboratory (mean reduction of 8 minutes), having a single call to a central page operator activating the catheterization laboratory (mean reduction of 14 minutes), activating the catheterization laboratory while the patient is being transported to the hospital (mean reduction of 15 minutes), expecting catheterization laboratory staff to arrive within 20 minutes after being paged (mean reduction of 19 minutes versus after 30 minutes), having an interventional cardiologist staying in the hospital (mean reduction of 15 minutes), and having the emergency department and the catheterization laboratory using real-time data feedback (mean reduction of 9 minutes) [19]. Another important aspect of these efforts to reduce D2B time is the commitment by the hospital administration to provide the support necessary for the implementation. In addition, close collaboration among the catheterization laboratory personnel, including the nursing staff, coronary care unit personnel, and the emergency room staff, is extremely important for the successful execution of these efforts. Adoption of these strategies can thus result in a significant reduction in D2B time, with anticipated improvement in clinical outcomes. A recent analysis of the Medicare patients showed a significant reduction in the median time to primary PCI from 96 minutes in 2005 to 64 minutes in 2010, and the majority (91.4%) with D2B time <90 minutes in 2010 versus 44.2% in 2005 [20]. Recent large-scale studies suggest that, for patients with STEMI presenting initially to hospitals without PCI capability, longer D2B times up to 120 minutes could provide excellent outcomes despite not receiving thrombolytic therapy at the non-PCI facility and undergo interhospital transfer to nearby PCI centers for primary PCI, although the goal should always be to minimize the D2B time [21–26].

 Unfortunately the majority of patients with STEMI cannot be treated with primary PCI due to the unavailability of PCI-capable hospitals in remote areas. It is estimated that primary PCI is available in <25% of hospitals in the USA [27]. On the other hand, nearly 80% of the US adult population lives within 1-hour drive of a PCI center [28]. Therefore, innovative ways to facilitate STEMI patient care at PCI centers could dramatically increase the number of patients treated with the preferred therapy of primary PCI. There have been several system-wide approaches to increase the number of patients treated with primary PCI [24, 29, 30]. One approach is the bypass model, which relies on prehospital ECG diagnosis of STEMI usually by the ambulance crew and supervised by physicians and transports the patient to the nearest PCI centers by bypassing closer non-PCI centers [29]. The Boston EMS Bypass STEMI Triage Plan and Treatment Registry is a prime example, although there are other cities with similar protocols more recently. The establishment of such a citywide STEMI treatment plan relies on the cooperation among the involved hospitals, especially the non-PCI centers, and standards as well as uniform data collection and outcomes monitoring. Although there may be false positive readings of the ECG, this approach does decrease the contact to balloon time [29]. Second approach is the transfer model, in which patients presenting to non-PCI centers are promptly triaged and transferred to the PCI centers using established protocols and integrated hospital systems [24]. The prototype of this transfer model is the Minneapolis Heart Institute's regional system, where 30 hospitals up to 210-mile radius from a PCI center used a standardized PCI-based treatment protocol for STEMI patients. Despite the logistic difficulties, the medial first D2B time for 60 to 210 miles was still 120 minutes, and the in-hospital mortality was 4.2% despite many high-risk patients [24]. The third possibility is to allow primary PCI in hospitals without onsite surgery [30], as studied in the C-PORT (The Atlantic Cardiovascular Patient Outcomes Research Team) trial [31]. The latter trial despite being underpowered showed that, following an appropriate training program, primary PCI without onsite surgery can be achieved with a shorter hospital stay (4.5 versus 6.0 days; *P* = 0.02) and reduced composite clinical endpoint of death, reinfarction, or stroke up to 6 months (12.4% versus 19.9%; *P* = 0.03) versus thrombolytic therapy. Importantly, no emergency coronary artery bypass surgery was required for PCI-related complications. With the availability of stents to treat dissections effectively to maintain vessel patency and potent pharmacologic regimen even for thrombus burden, currently primary PCI is rarely associated with emergencies. Obviously, these centers without on-site cardiac surgery should have a working relationship with a tertiary center for emergency transfer should the patients experience complications not corrected by the interventional and pharmacologic therapies.

But when these measures cannot guarantee acceptable D2B time or if there are no nearby PCI centers with high PCI volume or experienced interventional cardiologists, thrombolytic therapy despite its shortcomings is the preferred initial therapy, as some form of reperfusion therapy is more important than not providing any [32]. In addition, recent studies suggest that clopidogrel, an oral antiplatelet drug, administered with thrombolytic therapy can improve the patency rate of the infarct-related artery and reduce ischemic complications [33, 34]. Pretreatment with clopidogrel significantly increased TIMI flow, reduced infarct-related artery reocclusion, and improved survival without an increase in major bleeding versus aspirin alone.

## 3. Options following Successful Thrombolysis

If primary PCI is not an option and thrombolysis is administered, there are many different strategies for further therapy following successful thrombolysis, as the latter alone can still be associated with high reocclusion rates and reinfarction due to the residual severe stenosis in the majority of patients [13]. The important issue is whether these patients should be treated conservatively and thus undergo revascularization only if they have spontaneous (recurrent) ischemia or induced ischemia during subsequent stress test or whether all patients should be transferred to a PCI-capable hospital so that they undergo routine coronary angiography and revascularization if the anatomy warrants it. Regarding this dilemma, there have been randomized trials and meta-analyses to identify the optimal approach. These meta-analyses suggest that routine early PCI following thrombolytic therapy confers a significant reduction in the composite endpoint of death, reinfarction, and ischemia during the first year after STEMI without an increase in adverse events such as stroke or major bleeding [35–38]. Therefore, it is beneficial for patients after thrombolysis to be referred for routine coronary angiogram and revascularization, usually between 2 and 24 hours [39, 40]. TRANSFER-AMI (The Trial of Routine Angioplasty and Stenting after Fibrinolysis to Enhance Reperfusion in Acute Myocardial Infarction) showed that PCI within 6 hours after thrombolysis by immediate transfer to a PCI center following thrombolysis can significantly improve the composite endpoint of death, reinfarction, recurrent ischemia, new or worsening congestive heart failure, or cardiogenic shock within 30 days versus “standard” treatment of rescue PCI if necessary or ischemia-guided delayed angiography (11% versus 17.2%, *P* = 0.004) [41]. There was no increased major bleeding with the early routine PCI strategy.

## 4. Rescue PCI

 Since thrombolysis can provide successful reperfusion in only 50–60% of patients [13], rescue PCI is necessary in those with ongoing ischemia to minimize the infarct size and subsequent left ventricular dysfunction. As can be predicted, randomized trials clearly showed the benefit of rescue PCI, with 35% reduction in mortality and 36% reduction in reinfarction versus conservative approach [60]. In the largest study, the Rapid Early Action for Coronary Treatment (REACT) trial, patients randomized to rescue PCI had significantly improved primary composite endpoint of death, reinfarction, stroke, or severe heart failure within 6 months compared with repeated thrombolysis or conservative management (13.8% versus 25.6% with repeated thrombolysis and 22.4% with conservative management) [61].

## 5. Facilitated PCI

 An attractive option bridging the pharmacologic therapy and primary PCI is facilitated PCI, which refers to planned immediate PCI following an initial pharmacological regimen, usually thrombolytic therapy (full-dose or half-dose with or without a glycoprotein IIb/IIIa inhibitor) [62]. The hypothesis is that the pre-PCI pharmacotherapy can improve infarct artery patency and possibly earlier reperfusion. Unfortunately, no trial has shown a benefit [62], including two large trials, the ASSENT-4 (The Assessment of the Safety and Efficacy of a New Treatment Strategy for Acute Myocardial Infarction-4) PCI trial that had to be terminated prematurely due to increased mortality in the facilitated PCI group [63] and the FINESSE (Facilitated Intervention with Enhanced Reperfusion Speed to Stop Events) trial that evaluated the combination of abciximab plus half-dose reteplase or abciximab alone prior to PCI versus primary PCI [64]. Likewise, the LIPSIA-STEMI (Leipzig Immediate Prehospital Facilitated Angioplasty in ST-Segment Myocardial Infarction) trial [65] that compared facilitated PCI using tenecteplase versus primary PCI in STEMI patients presenting <3 hours after symptom onset and long transfer distances showed no benefit in infarct size as measured by delayed-enhancement magnetic resonance imaging. Therefore, facilitated PCI should no longer be considered in STEMI patients.

## 6. Recanalization of Occluded Artery beyond the 12-Hour Window

Those patients presenting beyond the 12-hour window after symptom onset and thus less likely to obtain myocardial salvage with intervention have been a dilemma regarding the recanalization of the occluded infarct-related artery. It has been estimated that up to one-third of patients with STEMI may not receive reperfusion therapy due to this late presentation [66, 67]. Whereas there is very little chance for myocardial salvage and there is in addition a procedure-related complication risk, there is also theoretical advantage of favorably affecting the left ventricular remodeling and provision of collateral source against future events [68–74]. As this group presented a dilemma regarding the optimal therapy, the OAT (Occluded Artery Trial) study randomized 2166 patients with total occlusion of the infarct-related artery 3 to 28 days after myocardial infarction and left ventricular ejection fraction <50% or proximal vessel occlusion to routine PCI and stenting with optimal medical therapy or to optimal medical therapy alone. The 4-year cumulative primary event rates were similar between the two groups, suggesting that routine PCI did not provide mortality or other cardiac benefit [55]. However, it is important to remember that OAT study excluded unstable patients, such as those in NYHA class III or IV heart failure, shock, angiographically significant left main or three-vessel coronary artery disease, angina at rest, and severe ischemia on stress testing. Subsequent analysis showed that those patients randomized to PCI had a higher risk of reinfarction due to reocclusion and stent thrombosis [75]. Thus, if the STEMI patients present >12 hours after the symptom onset and are clinically stable, routine angiography and angioplasty of the occluded infarct-related artery are not recommended.

## 7. Pharmacologic Advances

### 7.1. Antiplatelet Therapy

Platelets play a major role in the initiation and propagation of the thrombus during STEMI following plaque rupture. Therefore, antiplatelet therapy has been shown to reduce ischemic complications following STEMI [76]. Clopidogrel has been shown to be synergistic with aspirin in STEMI patients with thrombolytic therapy alone [33, 34] as well as PCI following thrombolytic therapy [77]. However, studies suggest that clopidogrel may not be the optimal oral ADP-receptor inhibitor due to a marked interindividual variability in its platelet inhibition [78]. Therefore, newer ADP-receptor inhibitors, including prasugrel [46] and ticagrelor [47], have been studied in STEMI patients compared with clopidogrel. In TRITON-TIMI (Trial to Assess Improvement in Therapeutic Outcomes by Optimizing Platelet Inhibition with Prasugrel-Thrombolysis in Myocardial Infarction) 38, prasugrel, a third-generation thienopyridine with improved pharmacodynamics and less interindividual variability when compared with clopidogrel [79], showed a significant reduction in major ischemic complications mainly due to the prevention of nonfatal myocardial infarction in patients presenting with acute coronary syndromes and treated primarily with stent implantation [46]. In addition, there was a significant reduction in stent thrombosis rate in the prasugrel group. This benefit, however, came at the expense of significantly increased bleeding risk. In addition, patients with previous transient ischemic attacks or stroke had worse outcomes when treated with prasugrel versus clopidogrel. Therefore, these patients have definite contraindication to prasugrel therapy. Furthermore, patients with low body weight <60 kg or age >75 years without high-risk comorbidities, such as previous MI or diabetes, also have relative contraindication to prasugrel therapy [46]. Finally, prasugrel was administered following coronary angiography and was not preloaded in majority of patients in the trial. Ticagrelor, a nonthienopyridine, direct acting, reversible P2Y12 receptor antagonist, also showed a significant reduction in ischemic complications, including cardiac mortality, versus clopidogrel in PLATO (Platelet Inhibition and Patient Outcomes) trial [47]. PLATO study also included patients treated conservatively without revascularization and showed similar benefit whether the patients were treated conservatively or with revascularization. Since neither mechanical nor pharmacologic reperfusion therapy is received by one-third of STEMI patients [66], the finding that ticagrelor improves survival in these “medically managed” patients is reassuring. Additionally, there was no subgroup with worse outcome versus clopidogrel except for those patients treated with >100 mg maintenance dose of concomitant aspirin. The only side-effects seen more frequently were the asymptomatic bradycardia and self-limited cough. Finally, there was no increased bleeding risk with ticagrelor therapy versus clopidogrel [47]. Therefore, given these two randomized trials showing the superiority of newer antiplatelet agents compared with clopidogrel, patients presenting with STEMI would benefit more from these agents following primary PCI versus clopidogrel.

### 7.2. Anticoagulant

Bivalirudin has been shown to provide effective anticoagulation during angioplasty with reduced bleeding complication compared with other options for both stable angina and low-risk acute coronary syndrome patients [80, 81]. Thus, the HORIZONS-AMI (Harmonizing Outcomes with Revascularization and Stents in Acute Myocardial Infarction) trial evaluated the beneficial role of bivalirudin in patients undergoing primary PCI with stenting versus unfractionated heparin and glycoprotein IIb/IIIa inhibitor therapy [48]. The trial showed a significant net clinical benefit, including markedly reduced bleeding complications and significantly reduced cardiac mortality in those patients randomized to bivalirudin therapy (9.2% versus 12.1%, *P* < 0.001). The benefits were maintained up to 3 years following the initial procedure [82]. One unexplained adverse outcome was the significantly higher acute stent thrombosis rate in bivalirudin group versus heparin plus glycoprotein IIb/IIIa inhibitor group although overall there were no clinical sequelae from this increased acute stent thrombosis risk. Therefore, bivalirudin should be considered as the preferred anticoagulant therapy for patients undergoing primary PCI.

### 7.3. GP IIb/IIIa Inhibitors

 Glycoprotein IIb/IIIa inhibitors have been shown to be beneficial in STEMI patients, especially when these patients were not pretreated with oral antiplatelet agents [83]. Recent studies suggest that intracoronary bolus administration of these agents rather than the traditional intravenous route may provide greater benefit as measured by infarct size and extent of microvascular perfusion and obstruction, especially in high-risk patients [84–86]. In addition, these trials provide the safety of the intracoronary bolus administration. Potential mechanisms include higher local platelet glycoprotein IIb/IIIa receptor occupancy and better post-PCI microvascular perfusion [87]. However, there have been inconsistent findings, especially with larger, randomized trials [88, 89]. Therefore, intracoronary bolus administration of glycoprotein IIb/IIIa inhibitor may be better reserved for high-risk patients. It has been shown that pre-PCI TIMI grade 3 flow of the infarct-related artery is an important positive predictor of improved survival [90]. The TITAN (Time to Integrilin Therapy in Acute Myocardial Infarction)-TIM 34 trial suggested that early (in the emergency room) versus late (in the cardiac catheterization laboratory) eptifibatide administration resulted in improved infarct vessel patency [91]. This study was a mechanistic study and underpowered to evaluate the clinical benefit of the early eptifibatide administration. Thus, early eptifibatide therapy may be considered in STEMI patients to increase spontaneous reperfusion. Although HORIZONS-AMI trial showed overall benefit of bivalirudin during primary PCI, an unanticipated finding of increased acute stent thrombosis rate was observed in the bivalirudin group versus heparin and glycoprotein IIb/IIIa inhibitor group [92]. Independent predictors of reduced acute and subacute stent thrombosis included prerandomization heparin and clopidogrel loading. Glycoprotein IIb/IIIa inhibitor therapy can be expected to provide protection against acute stent thrombosis, although prolonged infusion as has been studied in the randomized trials may cause bleeding complication. Thus, novel regimens, such as bolus only or shorter duration of infusion given the concomitant potent oral antiplatelet therapy, may reduce the bleeding risk while providing the upstream antiplatelet benefit [93]. Recent studies with prehospital administration of high-dose bolus tirofiban in the ambulance in addition to aspirin, heparin, and clopidogrel showed a significantly lower residual ST-segment deviation versus placebo [94], which resulted in a strong trend for decreased mortality at 30 days (2.2% versus 4.1%, *P* = 0.051) and 1 year (3.7% versus 5.8%, *P* = 0.08) as well as a significant reduction in major adverse cardiac events at 30 days compared with placebo (5.8% versus 8.6%, *P* = 0.043) [95].

## 8. Device

### 8.1. Drug-Eluting Stents versus Bare-Metal Stents

Bare-metal stents have become the device of choice during primary PCI, mainly by reducing target vessel revascularization without improving survival or reinfarction [96, 97]. However, observational studies suggested that there were possible safety concerns regarding drug-eluting stents versus bare-metal stents during primary PCI due to higher stent thrombosis risk [42, 98]. Subsequent randomized trials between the first-generation drug-eluting and bare-metal stents confirmed the benefit of drug-eluting stents in significantly reducing target-vessel revascularization [43–45]. In addition, there was no difference in mortality, reinfarction, or stent thrombosis, although the incidence of very late reinfarction and stent thrombosis was higher with drug-eluting stents [45]. The reason for this discrepancy of higher late stent thrombosis without associated higher mortality could be due to less catastrophic results of late stent thrombosis compared with acute or subacute stent thrombosis [99]. Therefore, selective use of drug-eluting stents in patients at high risk for restenosis without an increased risk of stent thrombosis may be warranted. It is unclear whether the second-generation drug-eluting stents will provide improved outcome versus the first-generation drug-eluting stents, especially regarding late stent thrombosis, as has been demonstrated in elective PCI [100]. One randomized trial between a first-generation DES (sirolimus-eluting stents) and a second-generation DES (everolimus-eluting stents) in STEMI patients showed a significant reduction of the major adverse cardiac events at 1 year (4.0% versus 7.7%, *P* = 0.048), with low one-year cardiac mortality (1.5% versus 2.7%, *P* = 0.36) and 1-year incidence of definite and/or probable stent thrombosis (1.2% versus 2.7%, *P* = 0.21) [101]. Although these results are encouraging, longer-term follow-up is necessary to confirm the definite advantage of the second-generation DES during primary PCI.

### 8.2. Distal Protection and Aspiration of Thrombus

Since STEMI patients are expected to have much thrombus burden and a high likelihood of distal embolization during primary PCI, it is logical to hypothesize that distal protection and thrombectomy devices would be beneficial. However, randomized trials have shown no benefit from distal protection during primary PCI [58]. Distal embolic protection devices did not reduce clinical events compared with PCI alone (3.1% versus 3.4% mortality). One possible explanation is the presence of multiple side branches in native coronary arteries, which cannot be protected with one distal protection device and may paradoxically have more distal embolization, especially with occlusion devices.

 Mechanical thrombectomy devices have not shown any benefit compared with no thrombectomy [58, 59]. On the other hand, manual thrombus aspiration using catheters has shown improved survival [49, 50]. Meta-analysis showed that catheter aspiration resulted in significantly lower mortality (2.7% versus 4.4%; *P* = 0.018) whereas mechanical thrombectomy resulted in higher mortality (5.3% versus 2.8%; *P* = 0.05) compared with standard PCI [58]. Although a recent randomized trial showed no benefit of manual aspiration [86], judicious use of manual aspiration devices may still improve outcomes during high-risk primary PCI.

### 8.3. LV Assist Devices

Intra-aortic balloon counterpulsation (IABP) has been shown to improve outcomes in patients presenting with STEMI and cardiogenic shock and thus has been recommended in these patients undergoing reperfusion therapy [102, 103]. New percutaneous ventricular assist devices have been studied in these patients and have been found to improve hemodynamics compared with IABP but may not confer mortality benefit [104, 105]. These novel devices may be beneficial if IABP support alone is not sufficient [105].

However, it is not clear if STEMI patients without cardiogenic shock may also benefit from IABP therapy. Therefore, the CRISP AMI (Counterpulsation to Reduce Infarct Size Pre-PCI Acute Myocardial Infarction) trial randomized high-risk anterior STEMI patients (at least 2 mm ST-segment elevation in 2 contiguous anterior leads or a total elevation of 4 mm or higher in anterior leads) to primary PCI or prophylactic IABP before primary PCI and continued for at least 12 hours and measured infarct size assessed by cardiac magnetic resonance imaging 3 to 5 days after PCI [56]. Patients with prior MI or CABG were excluded. In addition, bail-out IABP was allowed in the primary PCI only group if they had sustained hypotension or cardiogenic shock, uncontrolled arrhythmias, and acute mitral regurgitation or ventricular septal defect. The trial showed that there was no significant difference in infarct size between the two groups and there were no significant differences in clinical outcome. Although the study was not powered to show clinical difference, the results of this largest randomized trial in STEMI patients without shock suggest that IABP should not be routinely used in these patients.

## 9. Access Site

Due to the intense anticoagulation and antiplatelet therapy during STEMI treatment, bleeding complication used to be common, especially at the vascular access site [81]. Therefore, alternative access site such as the radial artery for primary PCI may provide a safer approach with reduced bleeding and, consequently, reduced ischemic complications, as major bleeding has been associated with increased risk of ischemic complications [106, 107]. Although there have been many observational studies suggesting this possibility [108], RIVAL (radIal versus femoral access for coronary intervention) trial was the first large, multicenter, randomized trial comparing the potential benefit of radial access versus femoral access in patients with ACS undergoing coronary angiography and intervention, including STEMI patients [51]. The trial found that although there was overall no significant difference between the radial versus femoral access groups, in patients with STEMI undergoing primary PCI, there was a significant benefit, including lower composite endpoint of death, recurrent myocardial infarction, or stroke, as well as death. Major vascular complications were also significantly reduced in the radial group. On the other hand, the access site crossover was significantly greater in the radial group. In addition, the highest tertile volume radial centers provided strong benefit whereas lower radial volume centers did not experience similar benefit. Likewise, HORIZONS-AMI trial showed that transradial approach resulted in reduced major bleeding and improved event-free survival versus transfemoral approach [52]. Recent meta-analyses of all randomized trials between transradial and transfemoral approaches in STEMI patients showed a significant reduction in mortality, major adverse cardiac events, and access site complications in the transradial group [53, 54]. On the other hand, there was only a trend toward a reduction in major bleeding, as some of the bleeding complications are from nonaccess site [51]. Thus, in high-volume radial centers, primary PCI via the radial artery access may reduce access-site vascular complications as well as overall ischemic complications during primary PCI versus transfemoral access.

## 10. Therapeutic Hypothermia

Many patients with STEMI experience out-of-hospital cardiac arrest as their initial presentation [109] and when these patients are successfully resuscitated and brought to hospitals, there are concerns about permanent hypoxic/anoxic neurologic injury despite successful reperfusion therapy with primary PCI. For these out-of-hospital cardiac arrest patients, mild therapeutic hypothermia from 32 to 34°C has been shown to improve neurologic recovery [110, 111], resulting in inclusion of hypothermia in the international guidelines on postresuscitation care [112]. Although induced hypothermia seems safe, the evidence for its efficacy is relatively weak [113, 114], and, thus, there is a need for further randomized trial to confirm the true benefit and ideal temperature range [115]. But until then, mild hypothermia in combination with primary PCI seems reasonable, as long as there are carefully planned executed protocols among the emergency room, cardiac catheterization laboratory, and intensive care unit staff [116]. Meticulous nursing care is also important to minimize the potential adverse effects.

## 11. Future Directions

### 11.1. Cell Therapy

Numerous studies have investigated the potential benefit of administration of bone marrow cells to improve left ventricular function in these STEMI patients, as even the most prompt reperfusion may not salvage all myocardium in jeopardy [117]. There have been positive studies, such as the REPAIR-AMI (Reinfusion of Enriched Progenitor Cells and Infarct Remodeling in Acute Myocardial Infarction) study, which showed a statistically significant improvement in ejection fraction with intracoronary bone marrow administered within 3 to 7 days of MI [118]. On the other hand, late administration such as 2 to 3 weeks after primary PCI (LateTIME randomized trial) did not result in increased global or regional ventricular function at 6 months [57]. In addition to the timing of intracoronary administration after myocardial infarction, the route of administration, such as intramyocardial delivery, may be important in improving the left ventricular function [119, 120]. Future studies will provide further guidance regarding the potential utility of bone marrow cell administration in patients presenting with STEMI [121].

### 11.2. Multivessel PCI versus Infarct-Related Artery PCI

One of the unresolved issues regarding primary PCI has been the treatment of significant lesions in non-infarct-related vessel during STEMI. It has been estimated that approximately 50% of patients with STEMI present with multivessel coronary artery disease [122–125]. These patients understandably have worse prognosis versus those with single-vessel coronary artery disease [126]. Despite the lack of a large randomized trial addressing the pros and cons of infarct-related vessel PCI only versus multivessel PCI in these patients, the latest guidelines stipulate that PCI of non-infarct-related vessels at the time of STEMI in hemodynamically stable patients is contraindicated with level III recommendation [127]. Recent retrospective studies [125, 128] as well as meta-analysis [129] suggest that simultaneous multivessel PCI in these patients during primary PCI was associated with higher mortality and stent thrombosis risk. One small but well-designed prospective randomized trial evaluating the different strategies in these patients (culprit vessel angioplasty only without further revascularization, staged revascularization at a later date, or simultaneous treatment of both infarct-related and non-infarct-related vessels) showed that the culprit vessel angioplasty only was associated with significantly higher in-hospital death, repeated revascularization and rehospitalization [130]. On the other hand, the other two groups had similar clinical outcomes up to 2.5 years. Analysis of a large New York State PCI Registry showed that multi-vessel PCI during the index hospitalization resulted in an increased in-hospital mortality compared with culprit-vessel PCI, but those patients undergoing staged multi-vessel PCI within 60 days after the primary PCI of the infarct-related vessel had a significantly lower 12-month mortality compared with culprit-vessel PCI only [131]. Until more definitive randomized trials are completed, it seems prudent to treat the infarct-related vessel only during the index procedure and stage the nonculprit severe lesions within 60 days after the primary PCI. In addition, the use of fractional flow reserve during staged procedure could identify the functionally significant coronary artery lesions more accurately versus angiography alone [132] and improve outcomes, including significant reductions in mortality and myocardial infarction [133] in these patients with multivessel coronary artery disease.

### 11.3. Novel Stents

Since drug-eluting stents may be associated with increased very late stent thrombosis risk versus bare-metal stents [45], novel stents with reduced stent thrombosis risk may be beneficial in STEMI patients. These advances can range from truly biodegradable [134, 135] to biodegradable polymer coating of the metallic scaffold [136] to stents coated with antibodies against circulating endothelial progenitor cells to enhance early endothelialization and reduced risk of stent thrombosis [137, 138]. Although these stents are still being developed for elective procedures, they could be especially useful for STEMI patients if their safety and efficacy can be demonstrated.

### 11.4. Need for Public Education and Faster Transfer for Primary PCI

It is accepted that the faster the revascularization after the symptom onset, the greater the likelihood of myocardial salvage and long-term outcome [139, 140]. Although D2B time is important, the symptom onset to balloon time is much more relevant regarding the infarct size reduction. Recent primary PCI trials showed that infarct size measured by technetium-99 m sestamibi single-photon-emission computed tomography imaging was the smallest when total symptom onset to balloon time was <2 hours and increased with longer time to reperfusion [141]. Therefore, it is extremely important that the public be educated on the symptoms of STEMI and be advised to seek medical care as soon as possible. One survey showed that the awareness of heart attack symptoms was low and did not improve from 2001 to 2007 [142], suggesting a need for more public education programs. In addition, women have been found to have both atypical symptoms [143] as well as higher risk of misinterpretation of symptoms [144]. Since many patients with STEMI still present to hospitals without on-site primary PCI capability and since transfer for primary PCI is still preferred if overall door to balloon time can be within the recommended time, the “door-in to door-out” (DIDO) time is important to salvage myocardium in these patients. However, recent study suggests that the acceptable DIDO is achieved in only a small percentage (<10%) nationwide [145]. Thus, new strategies to improve DIDO time are important. Finally, recent randomized, elective PCI studies in complex anatomy, such as left main coronary artery disease [146, 147], may provide the basis for more aggressive PCI strategy during STEMI for those complex lesions not treated with primary PCI thus far [148].

## 12. Conclusions

Recent advances in the management of STEMI patients (Table 1) have resulted in improved outcomes in this high-risk population. Whenever feasible, primary PCI should be offered. When primary PCI is not an option or cannot be performed in an efficient manner, thrombolytic therapy should be administered. However, these patients receiving thrombolytic therapy should be transferred to a PCI center sooner than later, and undergo angiogram and revascularization. Finally, an institution-wide, evidence-based pathway may improve adherence to pharmacologic therapies recommended by the guidelines and clinical outcomes [149]. 

## Figures and Tables

**Table 1:** Top ten advances in STEMI therapy.

Positive advances
Drug-eluting stents [42–45]
Potent oral antiplatelet agents [46, 47]
Bivalirudin [48]
Manual thrombus aspiration [49, 50]
Transradial intervention [51–54]

Negative findings

OAT trial [55]
CRISP AMI trial [56]
Stem cell therapy [57]
Distal protection [58]
Mechanical thrombectomy [59]
